# Coupling effects on turning points of infectious diseases epidemics in scale-free networks

**DOI:** 10.1186/s12859-017-1643-7

**Published:** 2017-05-31

**Authors:** Kiseong Kim, Sangyeon Lee, Doheon Lee, Kwang Hyung Lee

**Affiliations:** 1 Department of Bio and Brain Engineering, KAIST, Daejeon, South Korea; 2 Bio-Synergy Research Center, Daejeon, South Korea

**Keywords:** Epidemics, Social network structure, Scale-free, Susceptible-infected-recovered, Value of recovered on turning point, Spreading phenomena, Contagiousness, Recovery rate

## Abstract

**Background:**

Pandemic is a typical spreading phenomenon that can be observed in the human society and is dependent on the structure of the social network. The Susceptible-Infective-Recovered (SIR) model describes spreading phenomena using two spreading factors; contagiousness (β) and recovery rate (γ). Some network models are trying to reflect the social network, but the real structure is difficult to uncover.

**Methods:**

We have developed a spreading phenomenon simulator that can input the epidemic parameters and network parameters and performed the experiment of disease propagation. The simulation result was analyzed to construct a new marker VRTP distribution. We also induced the VRTP formula for three of the network mathematical models.

**Results:**

We suggest new marker VRTP (value of recovered on turning point) to describe the coupling between the SIR spreading and the Scale-free (SF) network and observe the aspects of the coupling effects with the various of spreading and network parameters. We also derive the analytic formulation of VRTP in the fully mixed model, the configuration model, and the degree-based model respectively in the mathematical function form for the insights on the relationship between experimental simulation and theoretical consideration.

**Conclusions:**

We discover the coupling effect between SIR spreading and SF network through devising novel marker VRTP which reflects the shifting effect and relates to entropy.

## Background

Epidemics, information, memes, cultural fads are representative spreading phenomena observed in human society. The pattern of spreading differs with the structure of the social network. SIR is one of models describing spreading phenomena suggested by A. G. McKendrick et al. [1] in 1924. The model expresses spreading in the form of differential equation among population compartments; susceptibles, infected, and removed. However, this model cannot reflect individual interactions. The network theory emerged since random graph model of Erdős–Rényi model (ER) [2] in the 1960s. Milgram showed the small world structure separated into 6 step distance through the experiment of mail forward which is reflecting interpersonal connection [3]. The interaction of each person can be represented by nodes and edges via network theory. There are three major network models of different features; scale-free network (SF) by Barabasi [4], small-world network (SW) by Strogatz [5] and ER random network. There are many types of research of spreading phenomena reflecting individual interactions through random network models. Keeling et al. [6] review of this research with the basis of epidemiological theory and network theory and suggest how the two fields of network theory and epidemiological modeling can deliver an improved understanding of disease dynamics and better public health through effective disease control. Shirley et al. [7] also compared epidemiological properties of some networks with different levels of heterogeneity in connectedness and mentioned that scale-free was fastest and reached largest in size, then random graph and the small world. Spreading phenomena are highly dependent on the network structure and understanding the structure is important to figure out and predict spreading phenomena. However, understanding the network structure is difficult because of its large scale, privacy, and difficulty in control. We devised a novel marker and observed changes in patterns of SIR epidemic spreading consequently on SF network model by simulation with various epidemic parameters and network parameters. It enables us to find an interesting aspect of coupling between the structure of the social network and the spreading phenomena.

### Network model

**Fig. 1:**
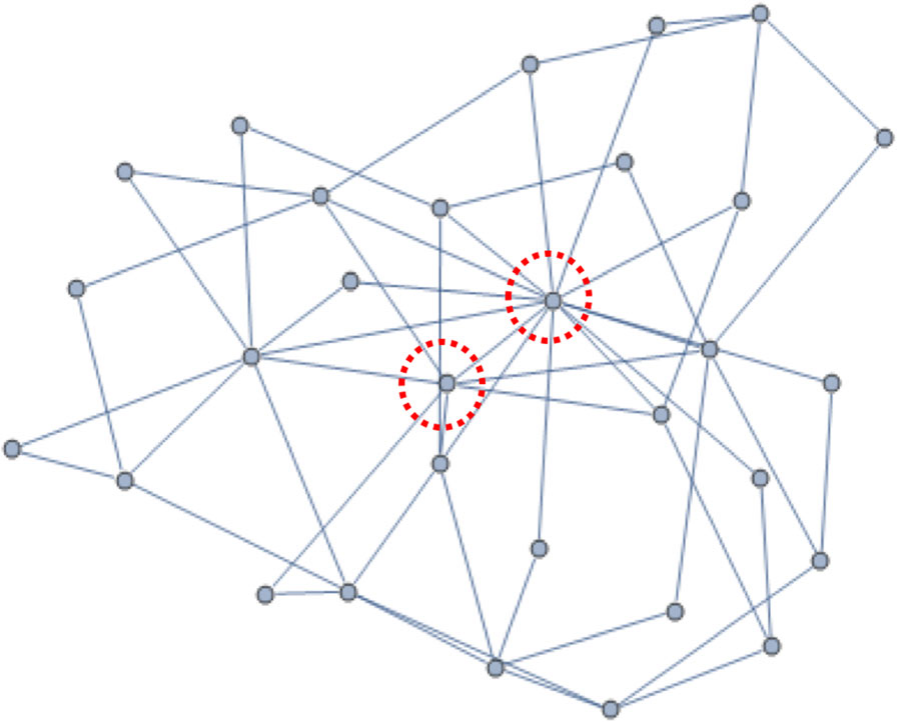
Scale-Free network with hubs. An example of a scale-free network. Highlighted two nodes are hub nodes, whose degree is larger than non-hub nodes

The scale-free model was suggested by A. Barabasi in 1999 [4]. This model shows fast, large-scale spreading because networks following this model are made up of many nodes of small degree and few nodes of large degree, “Hubs” (Fig. 1).

Many types of research on spreading phenomena used scale-free networks such as finding reproduction number [8], meme spreading [9] and analyzing computer virus data [10].

The small-world model was presented by Strogatz et al. in 1998 [5]. This network model has the possibility of the bridging link between distant nodes in spreading. Researches like percolation [11] [12] and transition to oscillation in epidemics [13] are based on the small-world network model. In this study, we used scale-free networks to reflect the fast and large-scale spreading.

**Fig. 2:**
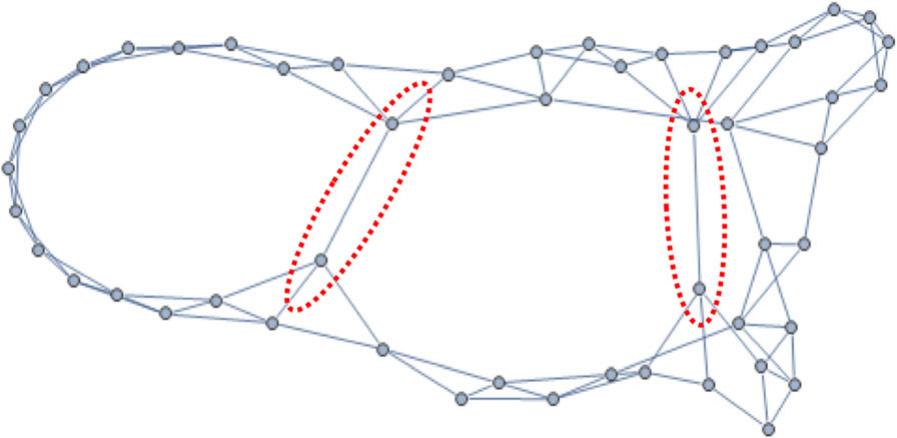
Small world network with bridging links. An example of small world network. Nodes in small world network are mainly connected with physically adjacent nodes. Based on probability, small world network has bridging edges which connect distant nodes. Two bridging edges are highlighted

Close nodes are connected each other while some bridging links which connect between far nodes appear (red stroke) (Fig. 2).

### Epidemic model

Many models are describing epidemic spreading. Those models are different in how they define population compartments. In this study, we used simple SIR model which considers two spreading factors; infection and recovery. SIR model uses three compartments; susceptible, infected, and recovered. In 1927, Kermeck and McKendrick presents three differential equation describing the relationship among three compartments.

1 $$ \frac{ds}{dt}= - \beta s i,\kern0.5em \frac{di}{dt}=\beta s i-\gamma i,\kern0.5em \frac{dr}{dt}=\gamma i $$

where *s*, *i* and *r* represents susceptible, infected, and recovered respectively [14]. Solving these nonlinear differential equations by the numerical approach, we can get the solution with the form of the time-series function of each compartment.

**Fig. 3:**
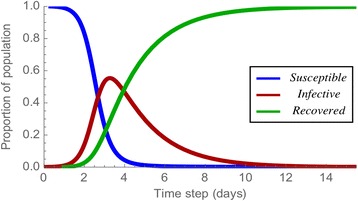
Change of epidemic populations in SIR. Time-series changes of the number of susceptible, infective, recovered nodes on SIR model. The blue, red, green curve shows the number of susceptible nodes, infective node, and recovered node respectively

Figure 3 shows the time-series change of the population of three compartments. The blue, red and green curve represent the change of Susceptible, Infective, and Recovered population respectively. The number of population of susceptible decreases steadily while population of infected increases in the early part and decreases after the turning point (TP) and population of recovered increases continuously. The Eq. (1) was modeled and derived on the assumption of the fully-mixed model, and it cannot reflect the epidemic spreading by the individual contacts.

**Fig. 4:**
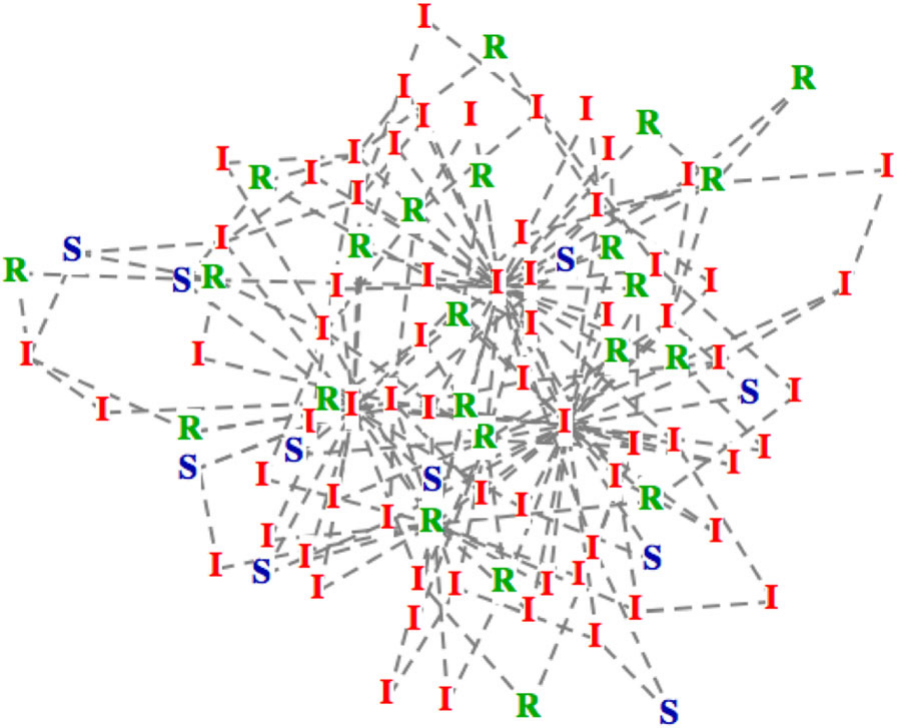
Example of an epidemic situation by applying SIR model to scale-free network. Snapshot of an epidemic spreading simulation on a network. Individual nodes are considered as people. Status of each people is expressed by character. S: susceptible, I: infective, R: recovered

In this study, as we applied the SF network model to our computational epidemic simulator, we devised a novel marker and observed the changes in each epidemic spreading in the networks. Figure 4 shows a snapshot of network spreading situation with the representation of each individual in compartments by using the graph theory with vertex and edge.

The blue “S”, red “I” and green “R” represent susceptible, infected, recovered individuals respectively, and the gray dashed line represents the contact relationship between individuals. Newman [9] calculated the SIR model solution in many different network models in his study. Trying to represent the real aspect of the social network, we used the scale-free network. We observed some features of the novel marker through the simulation of the epidemic spreading on SF network for different spreading parameters and network parameters.

## Methods

### Devising novel marker VRTP

**Fig. 5:**
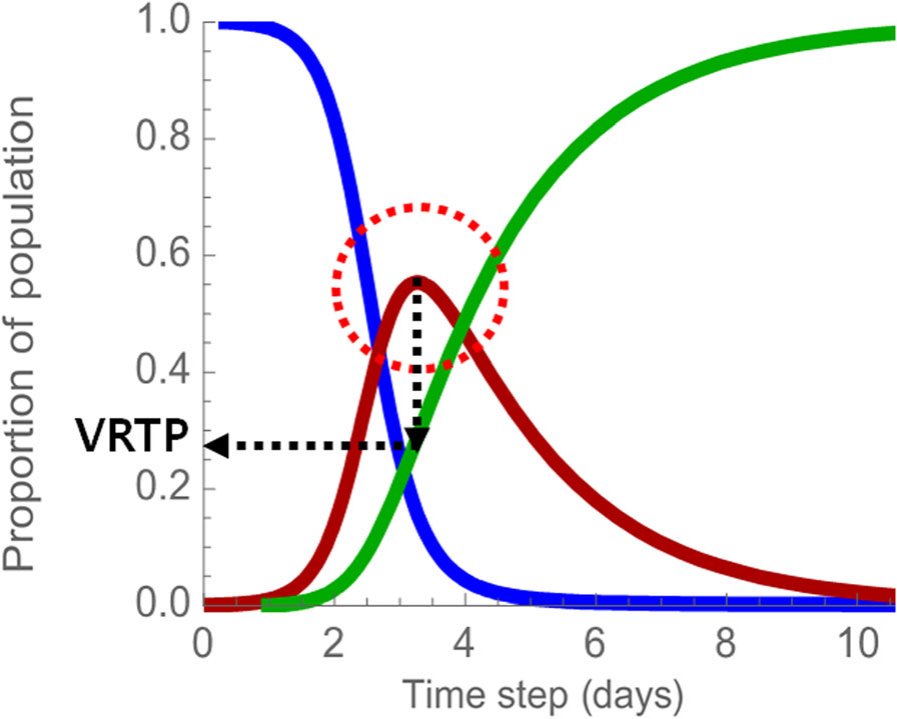
The value of marker, Value of Recovered at Turning Point (VRTP). The value of recovered at a turning point on SIR population graph. The number of recovered nodes when the number of infective node hits a peak

Here, we devise a marker VRTP which is the value of recovered population on the turning point. The turning point means the time point at the peak of the infected population. The turning point exists in the curves of the infected population in the SIR model. The number of the infected increases till the time is at turning point and after this point, the number of infected nodes decreases. We chose to observe the number of the recovered population as VRTP, the value of recovered population in turning point (Fig. 5). In general, many epidemics researches focused on the number of the infected population instead of the number of the susceptible and the recovered. As we see the relationship in the SIR model, the number of infected changes depending on the recovery parameter, which means that both curves are not independent. However, to understand and predict spreading in the social network in the other aspect, it is necessary to observe the number of S and R also. Through mathematical consideration and simulation for acquiring the maps of VRTP by the parameters, we found a coupling relationship between spreading parameter and network parameter.

### Epidemic simulation overview

**Fig. 6:**
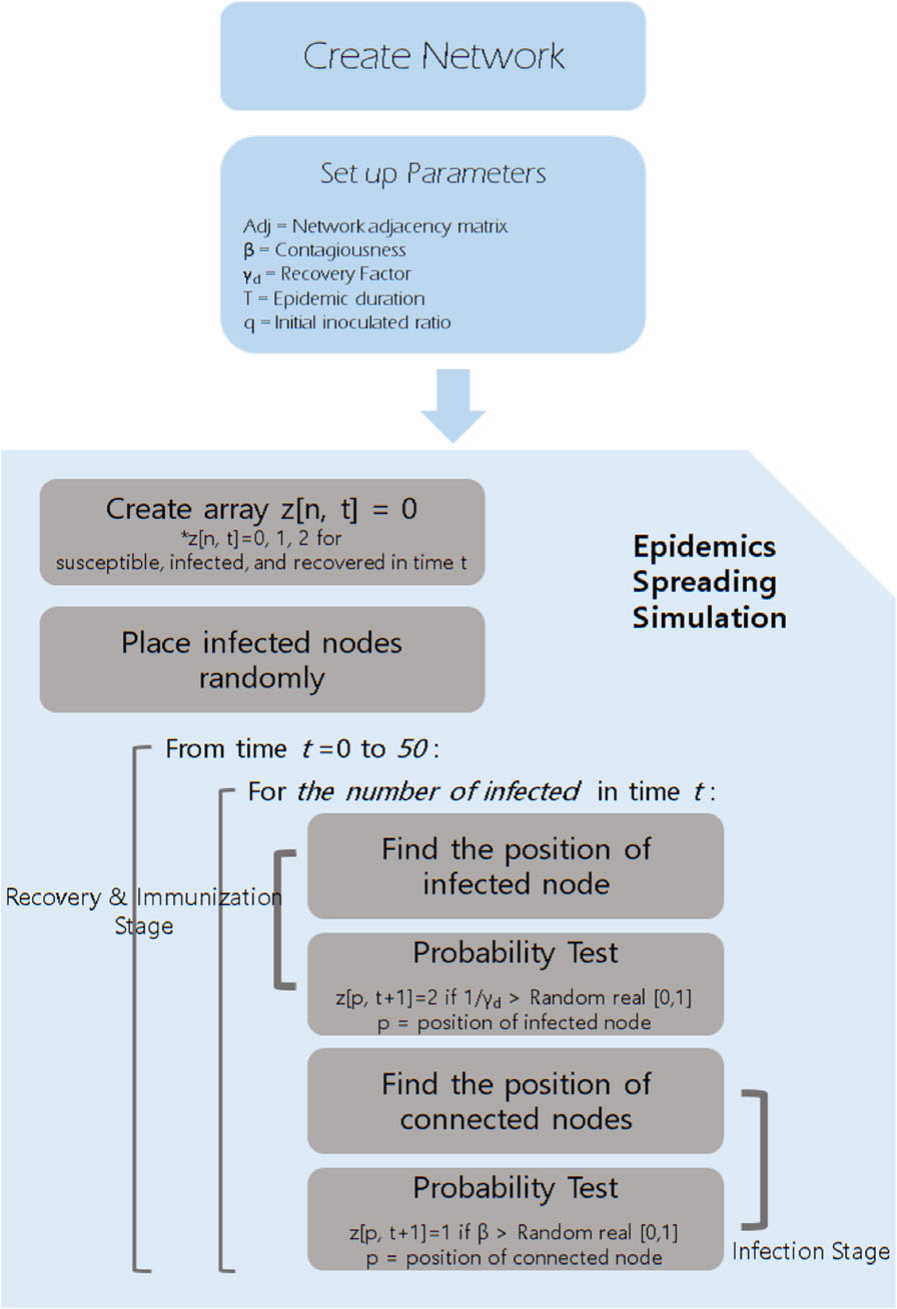
Overview of the epidemic simulation algorithm. The overall process of epidemic simulation algorithm. For network generated with certain parameters, we performed epidemic spreading simulation. After initial infection of random nodes, recovery stage and infection stage are repeated T (epidemic duration) times. Varying network parameter, we generated new networks and performed the epidemic simulation

We built an epidemic simulator for observing the spreading phenomena on the network. For the development of simulator and the simulation, we devise a simulation algorithm. The Inputs of the simulation algorithm include the network (adjacency matrix), *β* (contagiousness), *γ* (recovery factor), T (epidemic duration), and *q* (initial infect ratio). Epidemic duration T is the number of time steps of the simulation, and initial infection ratio *q* is the ratio of infective vertices to the whole population. First, it generates an SF network for simulation with given number of population and network parameter. It creates an array z, containing the status of the *n* ^*th*^ vertex in time *t*. For each vertex, the value of z at time *t* can be one of 0, 1, and 2 respectively represents that vertex is susceptible, infective, or recovered. We set every z to 0 at *t* = 0 because all vertices are susceptible before the epidemic spreading. It places the infective vertices randomly according to the initial infect ratio *q*. After the initial adoption, epidemic spreading simulation repeatedly works during epidemic spreading. Then the vertex falls into two kinds of random process stages, the recovery and immunization stage or the infection stage through contacts. In every cycle of epidemic spreading, firstly we search and find the infected vertices and then find infected nodes and their neighboring nodes. We adopted the Monte-Carlo probability experiment using *β* to determine whether the adjacent node becomes infected or not. In the recovery and immunization stage, through the Monte-Carlo probability experiment using *γ* again, we decide to make those infected vertices to be recovered or not. With this kind of process, the infected vertex in time *t* (z[*n*, *t*] = 1) become recovered vertex in time *t + 1* (z[*n*, *t + 1*] = 2) if *1/γ* is bigger than a random real number between 0 and 1. After this stage, in the stage of infection, we find susceptible vertices adjacent to infective vertices in time *t* (z[*n*, *t*] = 0, with the adjacent infective node). Among those susceptible vertices, a vertex becomes an infective vertex in time *t + 1* (z[*n*, *t + 1*] = 1), which represents epidemic transmission, if *β* is bigger than a random real number between 0 and 1. For each time step, we recorded the number of susceptible, infective, recovered vertices during epidemic spreading process (Fig. 6).

### Construction of VRTP distribution

From the result of the epidemic simulation, we can get the time series data of populations of each compartment. To make the time-series function smooth, we interpolate the discrete time-series data with the cubic spline function. Because the randomness exists in every trial of the epidemic simulation, we gather the result of every simulation trial by each parameter and calculate VRTP value in each trial and construct the distribution of the VRTPs to select the representative value. With Kolmogorov-Smirnov (K-S) statistical test [15], we figure out the distribution be the parametric Gaussian or not. Each mean value of VRTPs distribution from each simulation result by network and epidemic parameters was calculated for further analysis.

## Results

### Derivation of VRTP formula

For mathematical consideration of VRTP, we derive the VRTP formula with three theoretical assumption model. Those are the fully-mixed model (mean field model), the pairwise approximation model and the degree-based model.

For fully-mixed model based on ‘mass action principle’[16], we solved the nonlinear differential Eq. (1) algebraically remaining *r* at TP. The exact form of *r* at TP is like following,

2 $$ {r}_{TP}=\frac{ \ln \left({s}_0{R}_0\right)}{R_0} $$

where *r*_*TP*_ is VRTP and *R*_*0*_ is reproduction number, *s*_*0*_ is the initial value of the susceptible population.

Likewise, we solve the differential equations in the pairwise approximations model or moment closure method [17] (configuration model),

3 $$ <{r}_i\left( t= TP\right)>=\frac{ \ln \left(<{s}_i(0)>{R}_0{k}_i\right)}{R_0{k}_i} $$

where *k*_*i*_ is the degree of the i^th^ node.

And in the degree-based model [18],

4 $$ {\displaystyle \sum_{k=0}^{\infty }}{q}_k{r}_k(t)=\frac{ \ln \left({R}_0 k{s}_k(0)\right)}{R_0 k} $$

where *q*_*k*_ is the probability that a vertex with degree *k* is present.

**Fig. 7:**
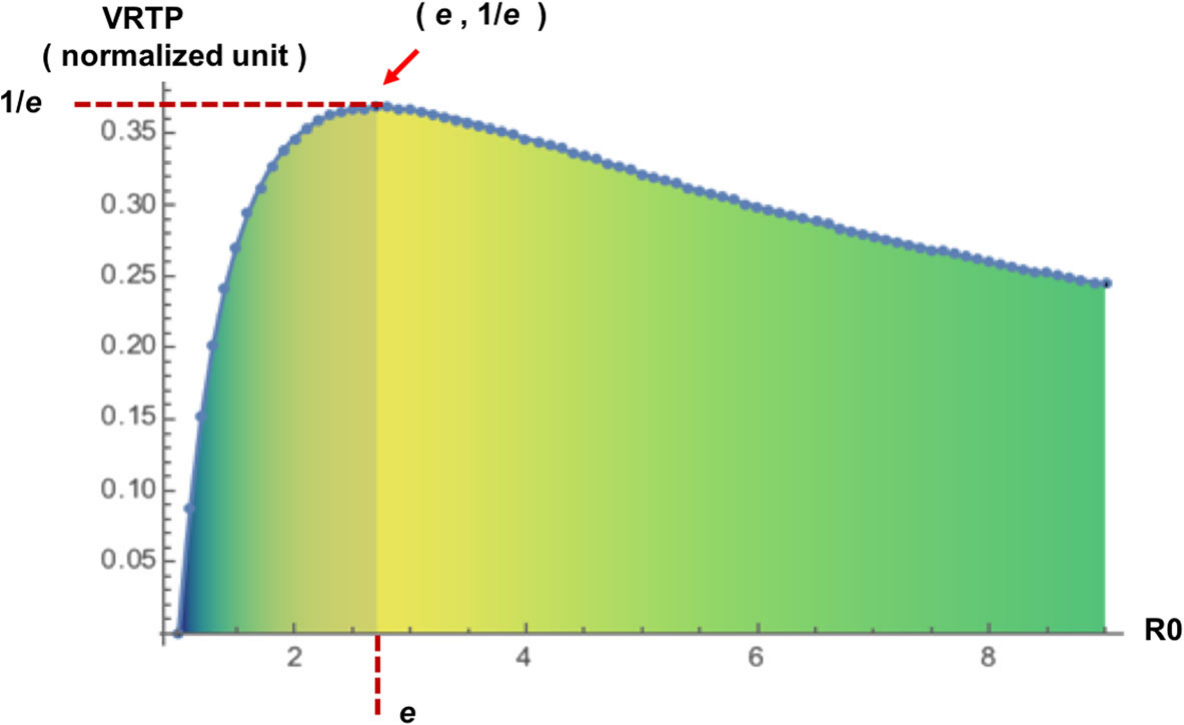
Theoretical VRTP values by reproduction number. The theoretical result which is calculated using the differential equation of SIR model without networks. The result of theoretical calculation shows the approximate range of VRTP, which is lower than *1/e* ~ 37% at *R*_*0*_ *= e*

As we can see, the whole form of the functions is like *ln(x)/x* for *s*_*0*_ ~ 1 and *x* is the reproduction number. The following figure (Fig. 7) shows the change of VRTP values by reproduction number.

From the result of theoretical consideration, finally, we can guess the range of the VRTP values in the SIR spreading model. In observation of the function form of VRTP, we can see that VRTP is low than *1/e* ~ 37% at *R*_*0*_ *= e*.

So, the range of VRTP is [0, *1/e*] and epidemic characteristics is divided into two regions, lower (*R*_*0*_ < *e*) and upper (*R*_*0*_ > *e*) region. The VRTP increases by *R*_*0*_ till the *R*_*0*_ *= e* and after that point, *R*_*0*_ decreases by *R*_*0*_. From the value of recovered population has the upper bound of 30% of the whole population when the infected population is maximum, we concluded that before the recovered being under 37%, and infected population would be decreased.

### VRTP surface and curves

As far as we know, the reproduction number *R*_*0*_ consists of two parameters, contagiousness *β* and recovery rate γ and specifically *R*_*0*_ *= β/γ*. We did the epidemic simulation by the parameters of two epidemic parameters, contagiousness *β* and recovery rate *γ* and of one network structure parameter *k*. From the simulation result, we constructed the distribution of VRTP and calculated the representative mean value of VRTP. Then we constructed the surface of VRTP and observed the change of VRTP varying those epidemic parameters.

**Fig. 8:**
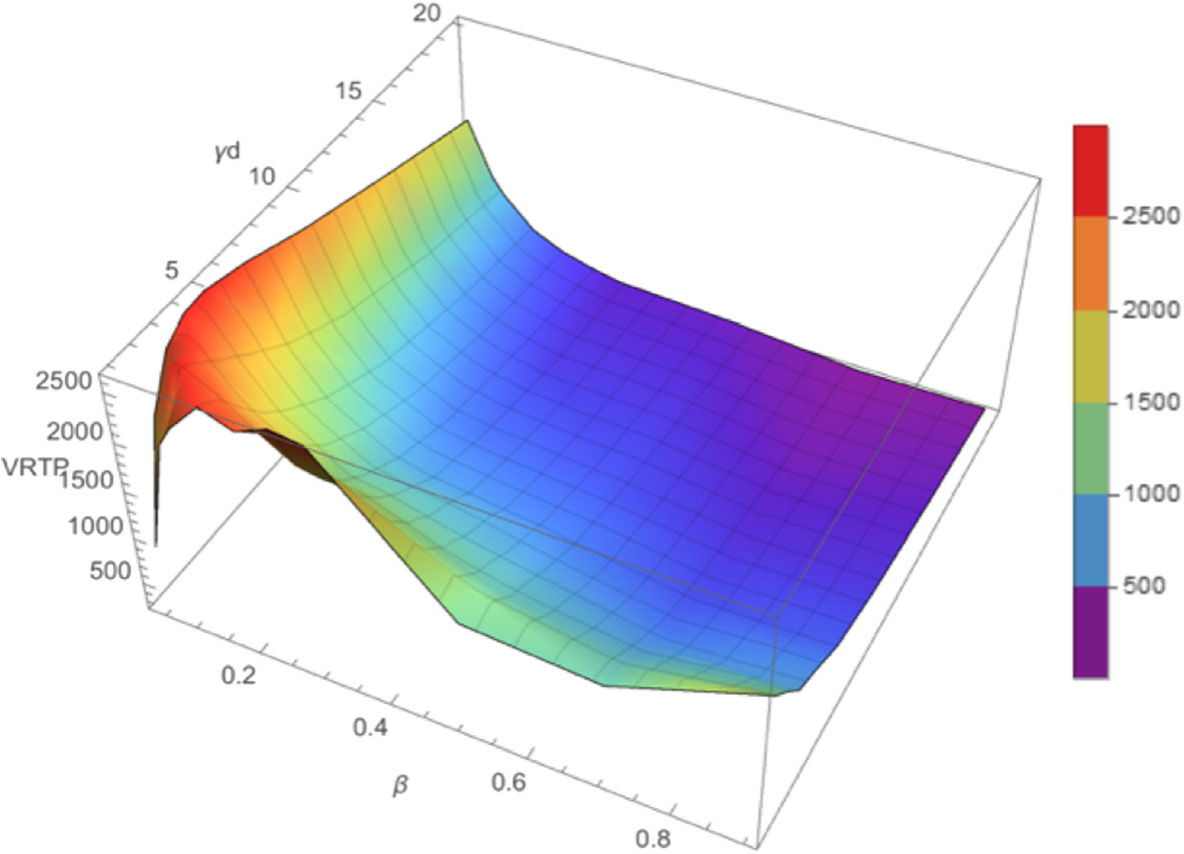
Example of VRTP surface. An example of VRTP surface. It shows some fluctuations while k increases, and the location of the peak of VRTP moves toward *β* = 0, *γ* = 0. VRTP always has its maximum value below about 30% of the whole population as we calculated theoretically

With both *β* and *γ*, VRTP increased rapidly from 0 to maximum value and decreased. The surface of VRTP shows some fluctuations while k increases, and the location of the peak of VRTP moves toward *β* = 0, *γ* = 0. Also, VRTP always has its maximum value below 30% of the whole population (Fig. 8).

**Fig. 9:**
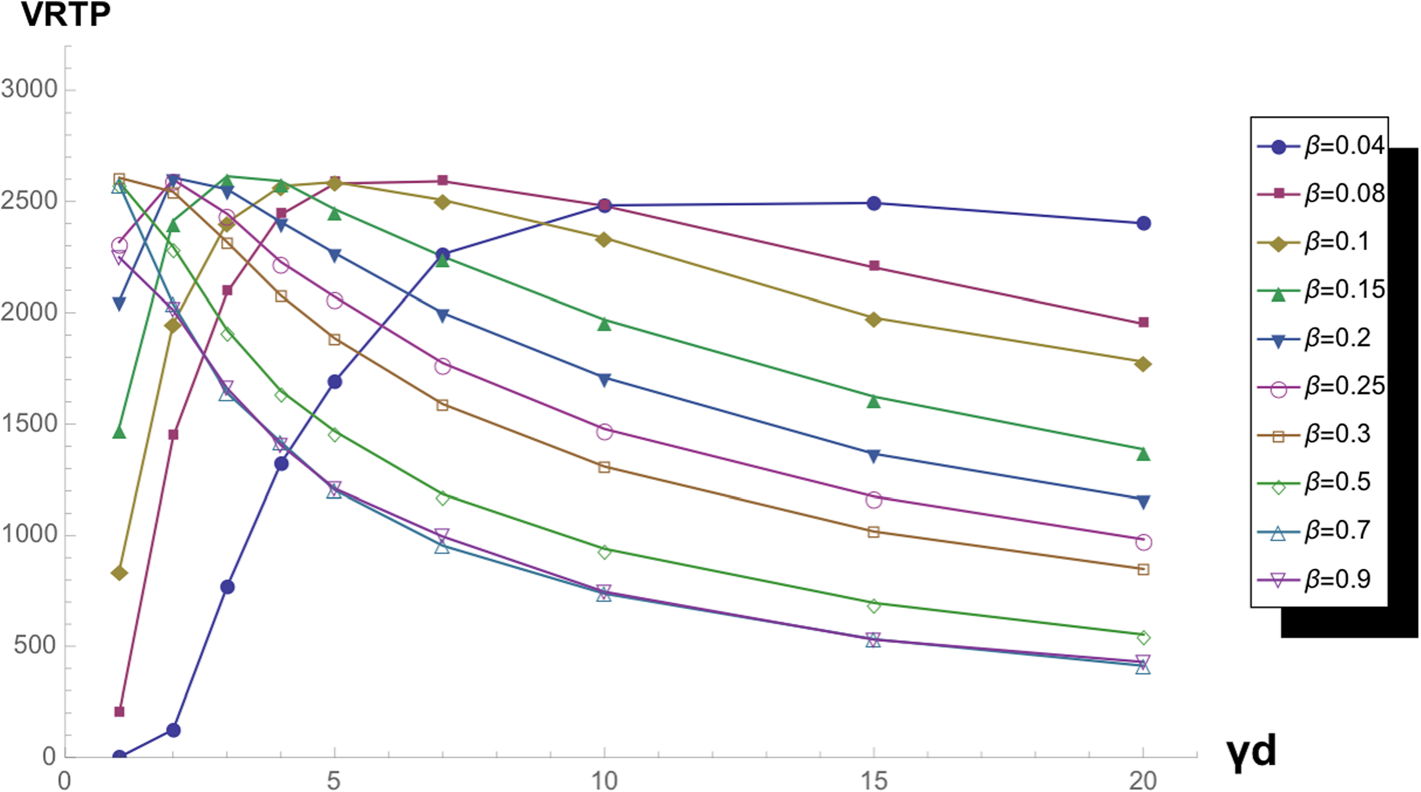
VRTP curves with varying *β,* in *k = 2* network. VRTP curve in k = 2 scale-free network varying *β* and fixed ***γ***_***d***_

**Fig. 10:**
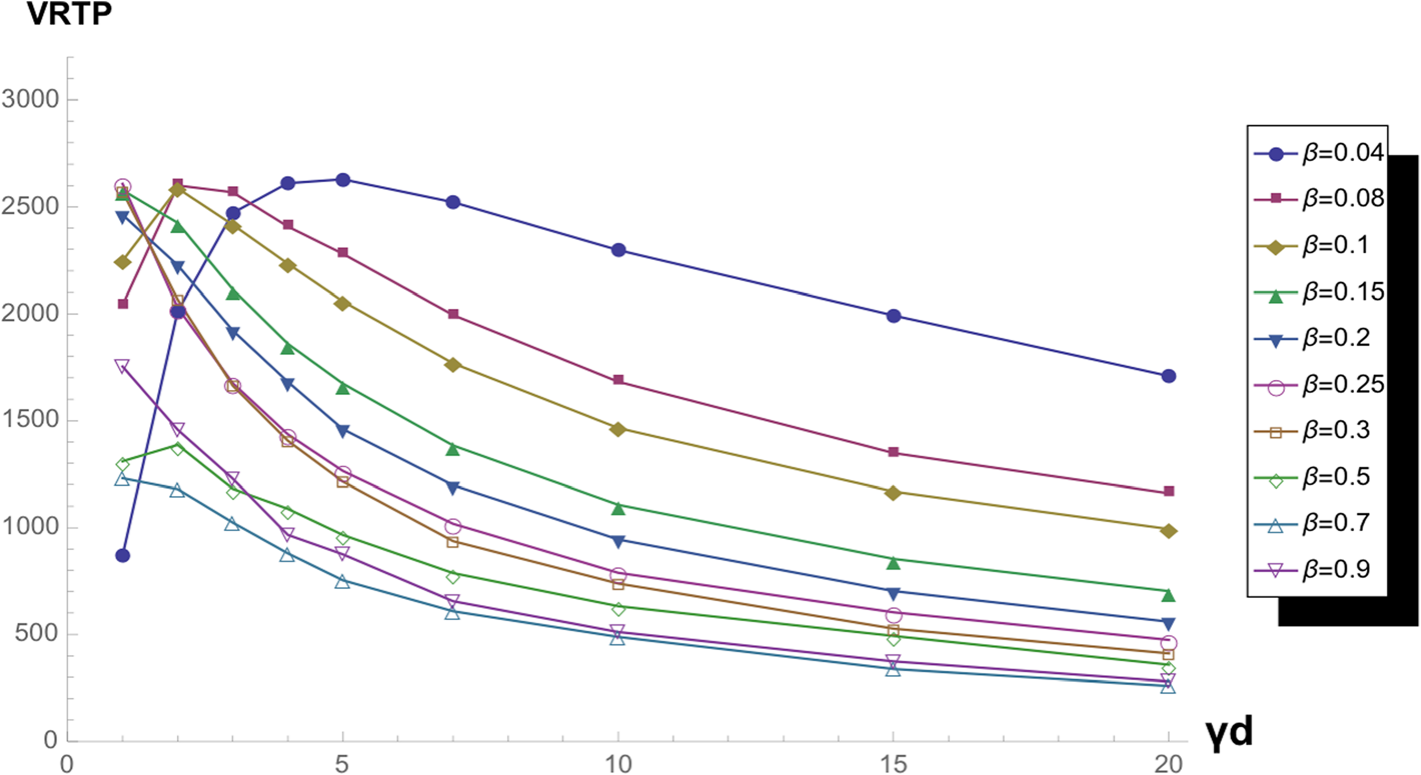
VRTP curves with varying *β,* in *k* = 10 network. VRTP curve in k = 10 scale-free network varying *β* and fixed ***γ***_***d***_

If we magnify the surface of *k* = 2 and *k* = 10, we can observe the area of the low beta area, we can see the smooth change between two curves of low *β* with sustaining same function form (Figs. 9 and 10).

**Fig. 11:**
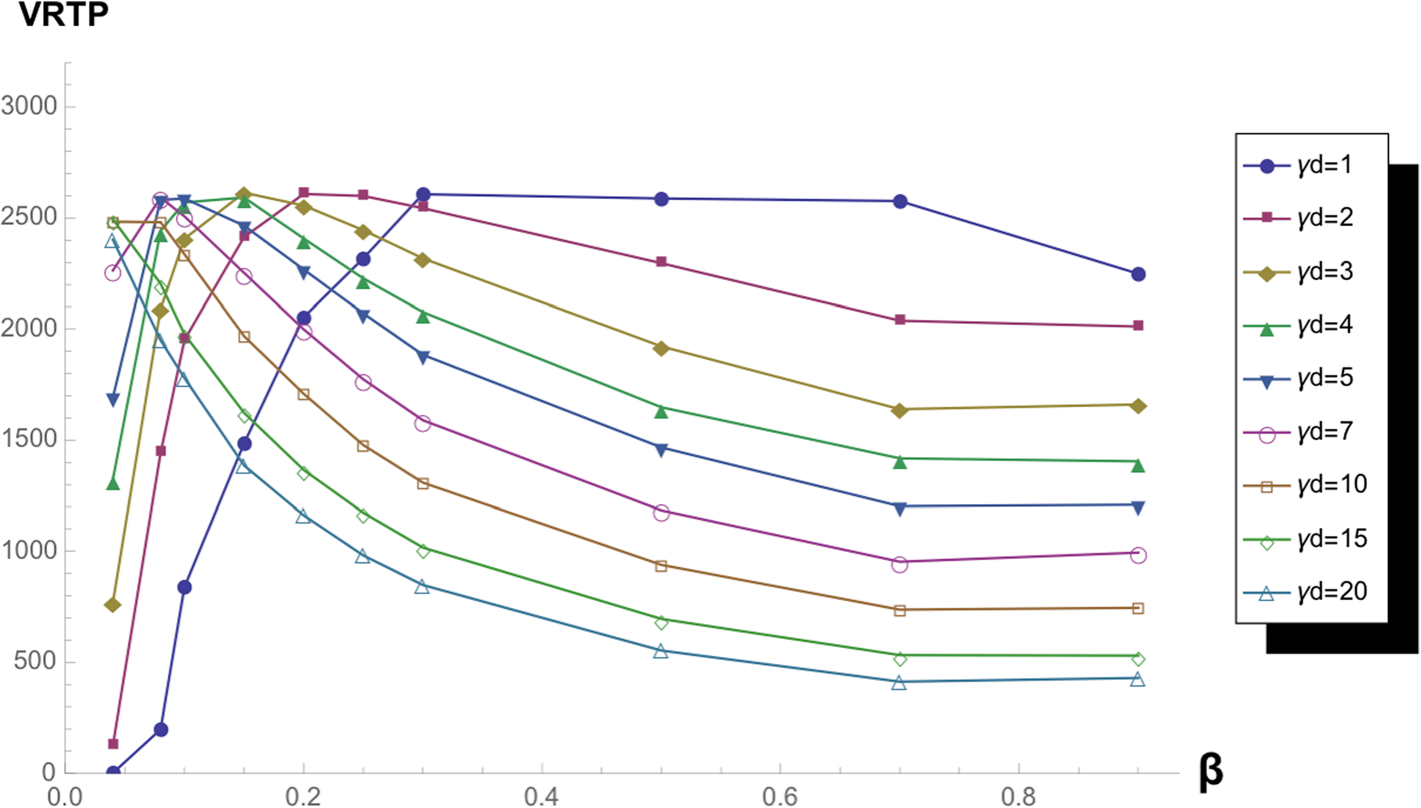
VRTP curves with varying *γ*_*d*_, in *k* = 2 network. VRTP curve in k = 10 scale-free network varying ***γ***_***d***_ and fixed *β*

**Fig. 12:**
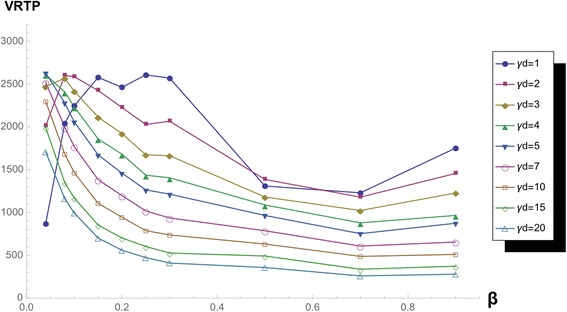
VRTP curves with varying *γ*_*d*_, in *k =* 10 network. VRTP curve in k = 10 scale-free network varying ***γ***_***d***_ and fixed *β*

With network parameter *k*, VRTP surface changes drastically in the region of low γ_d_. If we magnify the surface and observe the curve of low γ_d_ by *k*, we do not miss that the change between VRTP curves of low γ_d_ with changing the function form (Figs. 11 and 12).

**Fig. 13:**
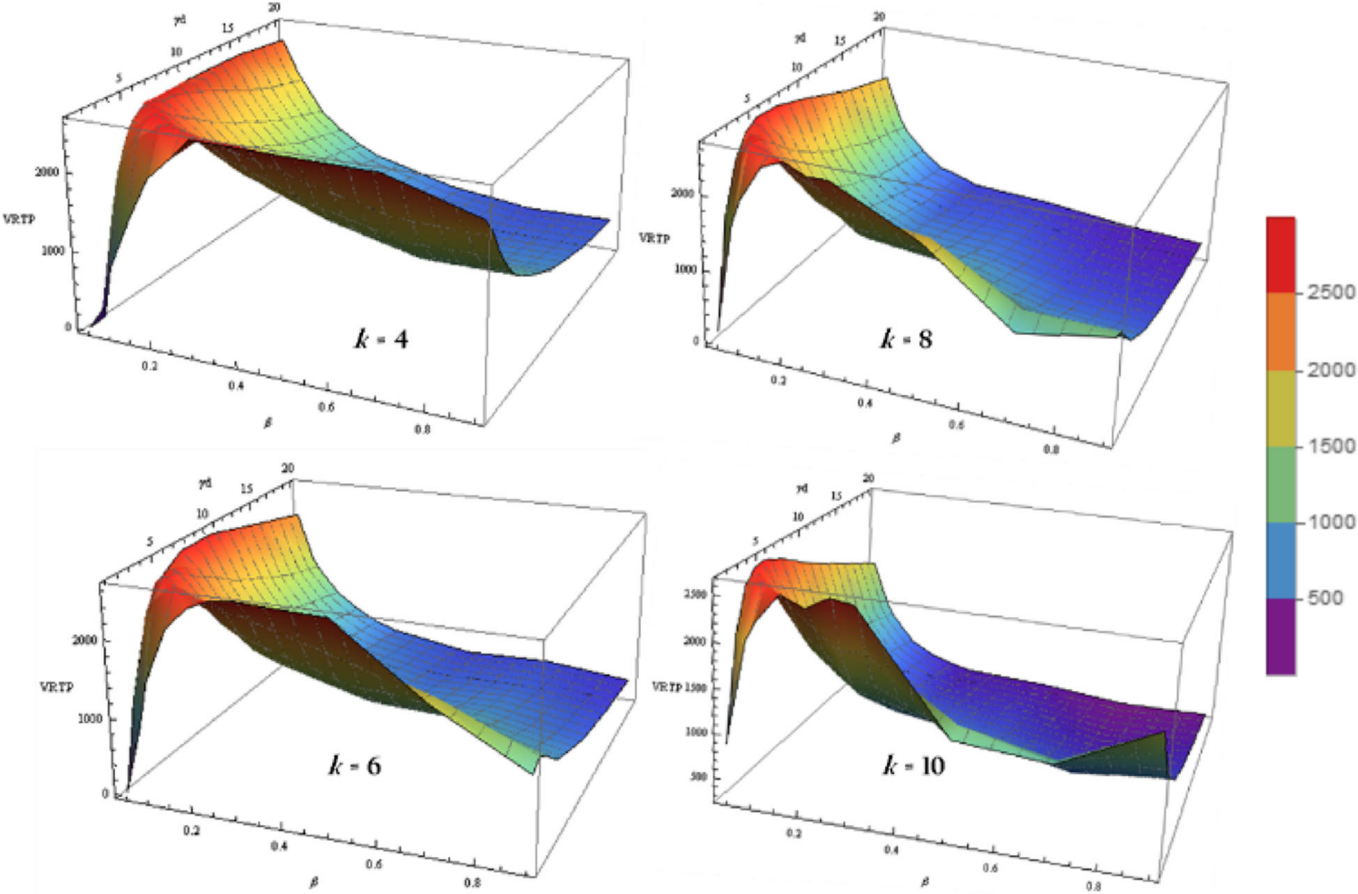
VRTP surface with varying *k.* VRTP curve in various scale-free networks varying k

We can observe same findings again in the VRTP surfaces with varying k (Fig. 13), which we find out in the previous curves.

## Discussion

### Derivation of VRTP formula Speed of spreading under fixed *γ*

k and *β* affect epidemic spreading speed under fixed *γ. β* is the probability of infection between the infective vertex and its neighboring susceptible vertex, so higher *β* results in the increase of speed. Likewise, parameter *k* used to decide the overall structure of network influences to the speed, vertices in networks with higher *k* shows higher network density than vertices in networks with lower *k*, which means more chances to spread epidemic to neighbors. *γ*_*d*_ is the inverse of *γ* and represents the recovery time after infection. Epidemics with high *γ*_*d*_ results in large number of infective vertices because they can stay in infective vertex for long time steps and infect neighboring susceptible vertices. These large *γ* cases are applicable to diseases like acquired immune deficiency syndrome (AIDS) in real-world. On the other hand, the number of recovered vertices increases rapidly in the event of epidemics with low *γ*_*d*_. In this case, the epidemic spreading is obstructed by recovered vertices because they are considered as disconnected vertices from the network. As a result, epidemics subsided because the recovery speed exceeds the speed of infection.

### Shifting the location of saddle point of VRTP curve

**Fig. 14:**
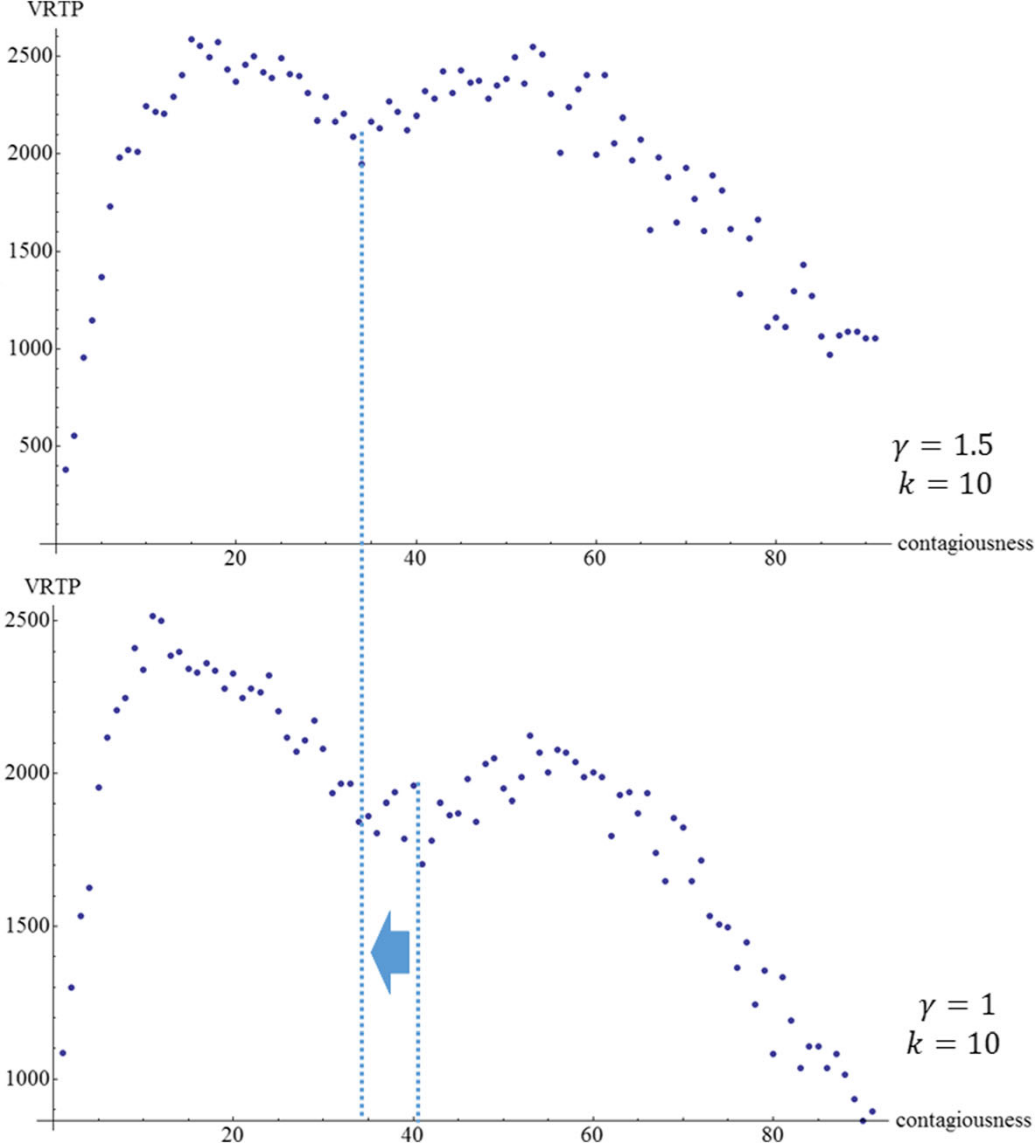
The location shift of the saddle point of VRTP curve along decreasing *γ.* The shifting of the saddle point of VRTP curve. Normally there are two peaks and a saddle point in VRTP curve. The curve is influenced by two factors, *γ* and (*β,k).* Changes of *β* and *k* increase the speed of epidemic spreading. As a result, the slight shift of VRTP curve to left happens. On the contrary, the increase of *γ* results in the shift to the right because the speed of spreading decreases

VRTP curves normally have two peaks and a saddle point (Fig. 14). With the observation of VRTP curves, we can conclude that two independent factors influencing the shape of VRTP curve, *γ* and *β-k*. Changes of *β* and *k* result similar effects. The increase of these values results in the faster spreading of epidemics. That makes a slight shift to the left of VRTP curve. On the contrary, the increase of *γ* results shift of VRTP curve to the right. Because the speed of epidemic spreading goes slower while connections of the networks become disconnected quickly as *γ* increases. However, VRTP curves with parameters *k* = 4 or larger *γ* do not show fluctuation. In the case of former, the speed of epidemic spreading becomes too slow to make fluctuation although *β* increases to 0.95. The latter case, in large *γ*, the obstructing power of *γ* by disconnecting vertices goes too weak to make fluctuation. We concluded that the saddle point appears to the point that satisfying 4 ~ *γ* × *β* × *k*.

### Network structure change by *k* and scaling effect

Eqs. (3) and (4) shows the characteristics of VRTP depends on *R*_*0*_ and also on *k* which is related the density of the network. In the case of low *k*, Eqs. (3) and (4) converse to Eq. (2). But in the case of high *k*, the *ln(x)/x* part can be considered as 1*/x*. And if we consider the effect of the network parameter, the reproduction number *R*_*0*_ should be adjusted as like *R*_*0*_ *k*. That is a scaling effect on VRTP. It is coincident with the result of Bartlett [19].

### Some aspect of VRTP function form

All of the VRTP formula has the function *ln(x)/x*. If we set *s*_*0*_ = 1 and substitute the inverse of reproduction number 1/*R*_*0*_ as the probability P, the function form would be the form − P ln(P). It is the form of Gibbs Entropy [20]. So we can infer that the VRTP may be related to the epidemic system information. And it is necessary for investigating more in the future work.

### Needs of the number of the recovered

There are not many epidemics spreading situations that we can draw the VRTP surface. For to draw the surface, we must know the VRTP values of whole epidemic parameters. But if get them, we figure out the network characteristics. We need the data which contains the time-series number of the recovered population with the infected population simultaneously. That makes us understand the characteristics of coupling effects in VRTP between the network and the SIR epidemics. For an example, in the prevalence of Influenza-Like-Illness (ILI), we must gather not only the data of the number of the infected but also of the recovered.

## Conclusions

We developed an epidemic simulator for the SIR spreading on the SF network. It has a handy ability to parameterize the epidemic processes, network types, node characteristics. And we devise the marker VRTP to reflect the epidemic turning points coupled to the recovered population and to discover the coupling effect between SIR spreading and SF network with the function form of the rough estimation among the parameters *k*, *γ*, *β*. We derive the analytic formulation of VRTP in the fully mixed model, the configuration model, and the degree-based model respectively in the form of entropy.

## Abbreviations

ER: Erdos-Renyi
ILI: Influenza-like-illness
K-S: Kolmogorov-Smirnov
SF: Scale-free
SIR: Susceptibles-Infectives-Recovered
TP: Turning point
VRTP: Value of recovered on turning point
